# 728 A Survey of Health Professionals' Attitudes Regarding Substance Abuse Disorder in Burn Patients

**DOI:** 10.1093/jbcr/irad045.203

**Published:** 2023-05-15

**Authors:** Karen Richey, Van Dobbe, Natalie Kesler, Steven Mouch, Kevin Foster

**Affiliations:** Arizona Burn Center Valleywise Health, Phoenix, Arizona; AZ Burn Center at Valleywise Health, Phoenix, Arizona; AZ Burn Center at Valleywise Health, Phoenix, Arizona; Arizona Burn Center Valleywise Health, Phoenix, Arizona; AZ Burn Center at Valleywise Health, Phoenix, Arizona; Arizona Burn Center Valleywise Health, Phoenix, Arizona; AZ Burn Center at Valleywise Health, Phoenix, Arizona; AZ Burn Center at Valleywise Health, Phoenix, Arizona; Arizona Burn Center Valleywise Health, Phoenix, Arizona; AZ Burn Center at Valleywise Health, Phoenix, Arizona; Arizona Burn Center Valleywise Health, Phoenix, Arizona; AZ Burn Center at Valleywise Health, Phoenix, Arizona; AZ Burn Center at Valleywise Health, Phoenix, Arizona; Arizona Burn Center Valleywise Health, Phoenix, Arizona; AZ Burn Center at Valleywise Health, Phoenix, Arizona; Arizona Burn Center Valleywise Health, Phoenix, Arizona; AZ Burn Center at Valleywise Health, Phoenix, Arizona; AZ Burn Center at Valleywise Health, Phoenix, Arizona; Arizona Burn Center Valleywise Health, Phoenix, Arizona; AZ Burn Center at Valleywise Health, Phoenix, Arizona; Arizona Burn Center Valleywise Health, Phoenix, Arizona; AZ Burn Center at Valleywise Health, Phoenix, Arizona; AZ Burn Center at Valleywise Health, Phoenix, Arizona; Arizona Burn Center Valleywise Health, Phoenix, Arizona; AZ Burn Center at Valleywise Health, Phoenix, Arizona

## Abstract

**Introduction:**

Substance abuse disorder (SAD) is complex, involving the continued use of drugs or alcohol despite harmful consequences. The diagnosis of this disorder carries a stigma that can bring shame to the individual, and healthcare providers are not immune to attitudes that worsen this shame. This coupled with patient behaviors can make caring for these patients challenging. We have seen an ever-increasing number of patients admitted to the burn center with this preexisting comorbidity. The purpose of this study was to better understand our own attitudes and potential bias or barriers that may be present in caring for this population.

**Methods:**

A modified version of the 11-item Medical Condition Regard Scale (MCRS) was distributed to burn center personnel via REDCap. An additional 6 burn specific questions were asked. Participants were asked to respond on a 6-point Likert scale. There were both positively (POS) and negatively (NEG) phrased questions. No personal or professional demographic data was collected to ensure the anonymity of respondents.

**Results:**

A total of 52 individuals responded to the survey. When responding to POS questions, the majority agreed that insurance coverage should be equal to other conditions (96%), would not mind being called in to care for this population (75%), felt they could find something to help the patient feel better (65%), felt compassion (65%), found working with SAD patients satisfying (65%) and enjoyed giving extra time to these patients (51%). When asked NEG questions, 40% responded it was difficult to work with these patients, 25% reported irritation, 23% preferred not to work with SAD patients, 13% felt there was little they could do to help, and 2% felt it was a waste of medical dollars. When asked POS burn specific questions, the sample was split 50/50 in regard to efficacy of current practice to manage withdrawal symptoms, 92% felt that these patients experienced more anxiety, had other mental health disorders (98%) and that pain management was particularly difficult (98%). When asked NEG burn specific questions 48% felt the care they provided was appreciated, and only 62% felt safe when providing care.

**Conclusions:**

The majority of staff reported willingness to help these patients yet reported difficulty in working with the population. Staff felt that these patients were more apt to have concurrent mental health disorders and increased anxiety. Management of pain and withdrawal symptoms were identified as areas of care that need improvement. It is concerning that 38% of staff felt unsafe in providing care. Identification of bias and barriers is the first step in an initiative to optimize protocols that will improve patient outcomes and work experiences.

**Applicability of Research to Practice:**

Protocols specific to the burn population that suffers from concurrent SAD are needed to optimize care.

